# Review of Magnetic Adsorbents for Heavy Metals in Sludge Leachate: Synthesis, Mechanism, and Performance Evaluation

**DOI:** 10.3390/ma19091691

**Published:** 2026-04-22

**Authors:** Shenglong Zhong, Shouming Hu, Ming Li, Xuyu Jiang, Jin Qi, Lihua Huang, Kai Zhu, Zongwei Xia, Nan Yu, Beibei Chen

**Affiliations:** 1 College of Resources and Environment, Linyi University, Linyi 276005, China; 2 Mengyin County Branch, Linyi Municipal Ecology and Environment Bureau, Linyi 276200, China; 3 Key Laboratory of Monitoring for Heavy Metal Pollutants, Ministry of Ecology and Environment, Hunan Ecological Environment Monitoring Center, Changsha 410019, China; xuyujiang2017@gmail.com; 4 Jinluo Water Management Co., Ltd., Linyi 276036, China; 5 Hunan Water Ecological Environment Monitoring Center, Hunan Ecological Environment Monitoring Center, Changsha 410019, China

**Keywords:** adsorption mechanism, heavy metals, magnetic adsorbents, performance evaluation, sludge leachate

## Abstract

The environmental challenges posed by heavy metal contamination in sludge leachate are becoming increasingly severe, necessitating the development of highly efficient remediation technologies. Among various treatment approaches, magnetic adsorbents have garnered significant attention as a promising solution due to their outstanding adsorption performance, convenient magnetic separation characteristics, and potential for regeneration. This paper systematically reviews the latest research progress on magnetic adsorbents designed for the complex system of sludge leachate, covering synthesis methods, surface functionalization, adsorption mechanisms, and performance evaluation. Key synthesis strategies are analyzed, including magnetic core preparation, inorganic coating, carbon composites, organic polymer grafting, functional molecule impregnation, and metal–organic framework (MOF) composites. The mechanisms by which these strategies influence material adsorption capacity, selectivity, and stability are elucidated. Despite significant achievements in laboratory studies, practical applications still face challenges such as large-scale synthesis, regeneration efficiency, cyclic stability, and adaptability to complex water bodies. Future research should focus on green synthetic pathways to advance the industrial application of structurally functional magnetic composite materials, providing systematic solutions from material design to process optimization for the sustainable remediation of heavy metal contamination in sludge leachate.

## 1. Introduction

With the rapid advancement of urbanization in China, sludge production has shown a sustained upward trend in recent years. Sludge treatment has become one of the most pressing environmental challenges requiring urgent solutions. Pollutants in water bodies originate from diverse sources, with various types of pollution introducing a wide array of contaminants into aquatic environments. These pollutants include both organic nutrients such as nitrogen (N), phosphorus (P), and potassium (K), as well as inorganic pollutants like heavy metals including Cd (II), Pb (II), Cr (III, VI), Cu (II), Ni (II), and Zn (II) [1,2]. Chromium primarily exists in two forms: Cr (III) and Cr (VI). Cr (III) is poorly soluble and has low mobility; it is an essential element for the human body and has low toxicity. Cr^6+^ is highly soluble and highly mobile; it is classified as a Group 1 carcinogen and is extremely toxic, primarily originating from industrial pollution [3]. Yang et al. found that the total concentrations of Cd, Pb, Cr, Cu, Ni, and Zn in municipal sludge from Nanchang City were 11.7 mg/kg, 113 mg/kg, 113 mg/kg, 383 mg/kg, 693 mg/kg, and 609 mg/kg, respectively [4]. If sludge is not properly treated and recycled, it can easily cause secondary pollution to the surrounding environment, with heavy metal contamination being a particularly prominent issue [5,6]. To better protect human health, water legislation aims to limit the maximum permissible concentrations of these metals to the lowest levels possible, depending on the type of water. For example, the World Health Organization’s interim drinking water guideline values for chromium, cadmium, lead, nickel, and copper range from 0.003 to 2 mg/L [7]. Their discharge levels in industrial wastewater are restricted to 0.01 to 0.5 mg/L.

Achieving safe disposal and resource recovery of sludge has become a hot topic in current environmental research. In the process of sludge safety treatment and resource utilization, leaching plays a crucial preliminary role. This process employs chemical solvents or biological methods to transfer heavy metals from the solid sludge phase to the liquid phase, thereby significantly reducing heavy metal content in sludge, mitigating its potential environmental impact, and creating favorable conditions for subsequent resource recovery [8]. Qiu et al. Experiments show that the maximum leaching rates of heavy metals Zn, Cu, Cd, Cr, Mn, Ni, As and Pb in municipal sludge within 10 days are 67.28%, 50.78%, 64.86%, 6.32%, 56.15%, 49.83% and 20.78, respectively [9].

**Figure 1:**
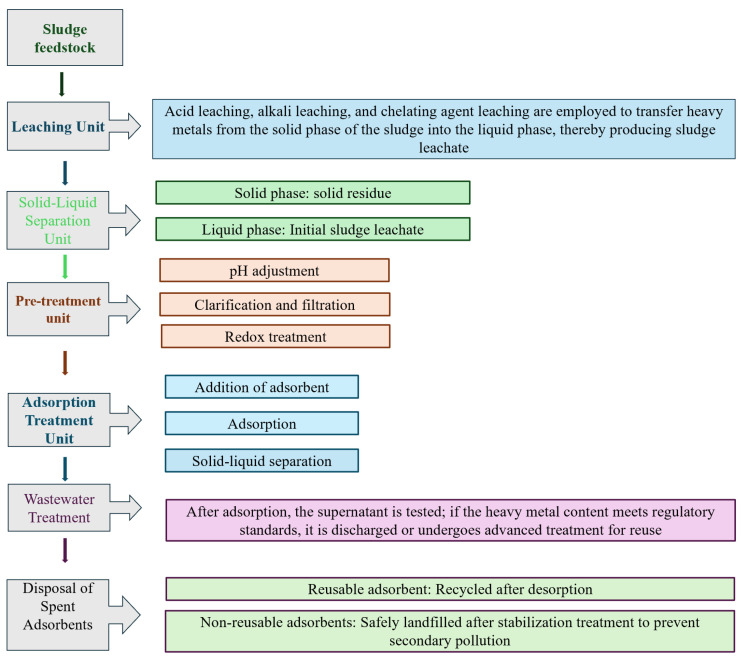
Flowchart of the Process for Treating Heavy Metals in Sludge Leachate Using Magnetic Adsorbents.

For the removal of heavy metals from sludge leachate (Figure 1), adsorption is considered the preferred method due to its advantages of simple operation, environmental friendliness, and high cost-effectiveness [10]. However, traditional adsorbents include activated carbon, clay, biochar, and pure sodium alginate (SA). Among these, pure sodium alginate (SA) suffers from limitations such as limited adsorption capacity, poor reusability, difficulty in separation, and insufficient mechanical strength. Furthermore, its synthesis process is often energy-intensive and inefficient, which limits its widespread application [11]. Activated carbon has a low adsorption capacity, limited selectivity, slow adsorption kinetics, and poor reusability [12]. Against this backdrop, novel magnetic adsorbents demonstrate significant application potential. Most “magnetic adsorbents” are not inherently single magnetic substances but rather composite materials composed of magnetic components and other functional constituents. The magnetic properties and separation efficiency of this type of magnetic composite are governed by several key factors, primarily including the synthesis method, the type and content of the magnetic phase, and the microstructure of the composite. These parameters directly determine the material’s magnetic separation rate and recovery efficiency, whereas the regenerative performance of the adsorbent depends mainly on the type of eluent, the binding strength of the adsorbate, and the desorption conditions; magnetic separation merely facilitates the rapid recovery of the adsorbent and does not directly affect desorption efficiency [13].

The development of magnetic adsorbents can be divided into five stages: initial exploration, technological foundation-laying, functionalization advancement, composite innovation, and accelerated industrialization. This progress has centered on three main threads: material preparation, performance enhancement, and application expansion. Robinson et al. were the first to apply magnetic materials to biotechnological separation, laying the theoretical foundation for magnetic adsorption separation [14]. Wu et al. established the standard protocol for the coprecipitation method, enabling precise control over particle size and magnetic properties, thereby laying the groundwork for subsequent research [15]. Giakisikli et al. systematically reviewed surface functionalization approaches and their applications from 2000 to 2013 [16]. Molaei et al. combined the ultra-high specific surface area of graphene with magnetic properties, discovering that it exhibits exceptional adsorption performance for heavy metals, organic compounds, and antibiotics [17]. Phouthavong et al. systematically summarized research progress regarding the green preparation of magnetic adsorbents, low-cost processes, engineering challenges, and corresponding solutions [18].

Current reviews on magnetic adsorbents primarily focus on heavy metal removal in single-phase aqueous systems; specialized reviews addressing the complex matrices of sludge leachates are scarce, and existing research findings are fragmented: Analyses of adsorption mechanisms are often based on a single characterization technique, lacking explanations of microscopic mechanisms derived from the combined use of multiple techniques. Furthermore, performance evaluation systems do not adequately account for selectivity, cycle stability, and engineering feasibility under complex matrices. There is a disconnect between laboratory research and practical engineering applications; issues such as the consistency of large-scale synthesis, the economic and environmental feasibility of regeneration processes, and the safe disposal of spent adsorbents have yet to be effectively resolved.

Based on this, this paper draws on research findings from core databases such as Web of Science, ScienceDirect, and ACS Publications from 2015 to the present. It focuses on studies of magnetic adsorbents in sludge leachate matrix systems, excluding low-value studies that lack explanations of adsorption mechanisms, quantitative performance data, or process innovations, while also incorporating classic foundational literature in the field. The paper establishes a systematic research framework covering “synthesis and preparation—functionalization and modification—adsorption mechanisms—performance evaluation”. First, this paper clarifies the classification criteria for magnetic adsorbents, organizing them according to core materials and surface functionalization pathways. Second, it systematically summarizes the synthesis methods and functionalization strategies for magnetic adsorbents, analyzing the regulatory mechanisms of each strategy on the material’s adsorption capacity, selectivity, and stability. Furthermore, by integrating advanced characterization techniques, it elucidates the adsorption mechanisms of heavy metals in sludge leachate at the molecular and atomic scales. Finally, it objectively analyzes the challenges faced by magnetic adsorbents in large-scale synthesis and engineering applications, and outlines future development directions, including green synthesis, computational modeling-aided design, and structure-function integration. This review aims to fill the gap in specialized research on magnetic adsorbents under the complex matrix of sludge leachate, providing systematic theoretical support and technical references for the targeted design and engineering applications of such materials.

## 2. Complex Matrix Characteristics of Sludge Leachate

Sludge leachate is the liquid byproduct of heavy metal leaching from sludge; its volume increases as sludge production increases, primarily due to improvements in wastewater treatment technology and the expansion of sewer systems [19]. Sludge leachate is not a simple system consisting of a single heavy metal ion; rather, it is a complex, multifaceted system characterized by the diversity of heavy metal forms, the complexity of coexisting substances, and the variability of the matrix environment. Its unique matrix properties not only directly influence the migration and transformation patterns of heavy metal ions but also impose stringent requirements on the adsorption performance, selectivity, and stability of adsorbents, making them the core considerations for the adaptive design and process optimization of magnetic adsorbents.

### 2.1. Characteristics of Heavy Metal Distribution and Mobility

The heavy metals in sludge leachate originate from the leaching process of the solid-phase sludge and are characterized by various heavy metals and coexisting substances, which can influence the adsorption process. In terms of heavy metal content, a variety of heavy metal ions have been identified, including Al (III), Ca (II), Cu (II), Fe (III), Mg (II), Ni (II), and Zn (II) [20]. Specific concentrations of certain heavy metals in the leachate have been reported: Cu 13.4 mg/L, Zn 9.9 mg/L, Cr 7.1 mg/L, as well as Ca (II) (32.9 mg/L), Mg (II) (2.2 mg/L), Pb (II) (0.001 mg/L), and Fe (III) (0.002 mg/L) [21]. This difference in concentration directly leads to intense competitive adsorption during the adsorption process.

The speciation and mobility of heavy metals are key properties influencing their behavior in leachate. Zhang et al. found that Cu and Cr primarily existed in the F_3_ form in the original sludge, with concentrations of 72.1 mg/kg and 47.7 mg/kg, respectively, accounting for 70% and 40% of the total metals. This indicates relatively low bioavailability and mobility. In contrast, Mn and Zn predominantly existed in the F1 form (water-soluble and carbonate-bound), with concentrations of 139 mg/kg and 150 mg/kg, respectively, accounting for 72% and 43% of total metals. This indicates high bioavailability and mobility [22]. Additionally, Heavy metals such as Zn, Cu, and Cd primarily exist in unstable forms within sludge systems. Following composite conditioning by Wang et al., the proportions of the five heavy metals in the filtrate were 3.16%, 6.76%, 0.88%, 1.73%, and 1.66%, respectively. Except for Cr, over 99% of the heavy metals remained in the filter cake, which affected the original distribution [23]. Heavy metal ions in different forms exhibit distinct adsorption response characteristics in leachate, which directly influences the direction of targeted adsorbent design.

### 2.2. Composition and Effects of Coexisting Interfering Substances

The co-existing substances in sludge leachate primarily originate from the organic matter and inorganic salts in the sludge itself, as well as chemical reagents introduced during the leaching process. Among these, high concentrations of ammonia nitrogen and resistant dissolved organic matter (rDOM) are the primary organic interferents, while inorganic salt ions are the main inorganic interferents. These coexisting substances interfere with the removal of target heavy metals by magnetic adsorbents through mechanisms such as competing for adsorption sites, altering the physicochemical properties of the solution, and forming complexes with heavy metals. Dai et al. found that coexisting substances in sludge leachate primarily originate from aged landfills, including high levels of ammonia nitrogen (>500 mg/L) and recalcitrant dissolved organic matter such as humic substances, lignin, and recalcitrant dissolved organic nitrogen. In mature leachate, rDOM concentrations typically approach COD values due to lower concentrations of biodegradable organic compounds [24]. High concentrations of NH_4_^+^ can compete with cationic heavy metals for adsorption sites; this competitive effect is particularly pronounced when electrostatic attraction is present at the adsorbent surface, directly reducing the adsorption capacity and removal efficiency of the target heavy metals. Boyle et al. were the first to discover that banana peel-based adsorbent beads exhibit a synergistic enhancement effect on the adsorption of tetracycline following the pre-adsorption of hexavalent chromium. They demonstrated that the adsorption active sites for different pollutants do not compete with one another, and that hexavalent chromium alters the chemical environment of the adsorbent beads, thereby improving the removal efficiency of tetracycline [25].

Magnetic adsorbents for sludge leachate cannot simply adopt the design principles used for ordinary water bodies; instead, they require targeted structural design and functional modification based on the complex characteristics of the matrix: By introducing specific functional groups to enhance selectivity toward target heavy metals, employing strategies such as inorganic coating to improve the material’s structural stability across a wide pH range, and utilizing composite modification to construct a multi-mechanism synergistic adsorption system, efficient removal of heavy metals in various forms and concentrations can be achieved. This also provides clear design guidelines and optimization objectives for subsequent magnetic adsorbent synthesis methods and functionalization strategies.

## 3. Synthesis and Functionalization Strategies for Magnetic Adsorbents

### 3.1. Characteristics and Preparation of Core Materials

Common magnetic core materials for heavy metal removal adsorbents include Fe_3_O_4_, γ-Fe_2_O_3_ and MnFe_2_O_4_. Fe_3_O_4_ and γ-Fe_2_O_3_ have highly similar structures (both are cubic inverse spinel); the key differences are as follows: Fe_3_O_4_ contains Fe (II) and Fe (III), has a complete structure, is strongly magnetic, and is conductive; γ-Fe_2_O_3_ consists entirely of Fe(III), contains cation vacancies, exhibits weaker magnetic properties, is insulating, and readily transforms into α-Fe_2_O_3_ at high temperatures; it possesses greater resistance to oxidation, and γ-Fe_2_O_3_ is more stable than Fe_3_O_4_ in acidic environments [26]. Fe_3_O_4_ is widely used due to its high stability, exhibiting superparamagnetism at the nanoscale and possessing abundant surface hydroxyl groups that can be protonated or deprotonated by adjusting pH to generate positive or negative charges [18]. Although magnetic iron oxide occurs naturally, we can synthesize it in the laboratory through various methods using coprecipitation [27], Hydrothermal [28], Solvent-thermal [29], and Thermal decomposition [30]. Various methods are employed to synthesize them in the laboratory, thereby controlling particle size and achieving higher purity. The process characteristics, advantages, disadvantages, and suitable applications of each method vary significantly; see Table 1 for a summary of the key differences. The coprecipitation method is the most widely used due to its simplicity and low-temperature operation. The solvent-thermal method offers the advantage of forming nanostructures with specific morphologies and high crystallinity, making it suitable for preparing magnetic materials with stringent structural and performance requirements, such as Fe_3_O_4_ nanoparticles used in highly active catalysts. However, this method requires specialized equipment, incurs higher costs, and involves prolonged reaction times, which hinder large-scale production.

Composites based on MnFe_2_O_4_ have a significantly higher specific surface area than Fe_3_O_4_, possess a greater abundance of active sites, exhibit higher adsorption capacities for heavy metals, and demonstrate superior thermal and acid-base stability compared to pure Fe_3_O_4_. Below 328 °C, they undergo only dehydration and weight loss; they remain stable in sludge leachates with pH values ranging from 3 to 8, and their surface functional groups are not easily degraded, making them particularly well-suited for acidic bioleachates [31]. Iron oxide nanoparticles synthesized via coprecipitation at different temperatures (25 °C, 60 °C, 90 °C) exhibit temperature-dependent characteristics: increased synthesis temperature enhances nucleation and reduces average microcrystalline size, while saturation magnetization (Ms) increases with rising temperature [32]. The specific surface area of the pure Fe_3_O_4_ magnetic nanoparticles prepared by Chen et al. was characterized as 182 m^2^/g [33], while that of the MnFe_2_O_4_ magnetic nanoparticles prepared by Sun et al. was 245 m^2^/g [34]. Magnetic nanoparticles (MNPs) exhibit high specific surface area, size-dependent catalytic activity, and ease of magnetic separation. During adsorption processes, they can achieve efficient adsorption through the formation of physical or chemical bonds [35]. These core magnetic materials and their preparation methods lay the foundation for developing magnetic adsorbents. Different preparation methods can endow materials with distinct properties to suit various application scenarios and requirements.

**Table 1:** Comparison of Primary Preparation Methods for Magnetic Nanoparticles.

Preparation Method	Advantages	Disadvantages	Typical Product	Suitable Scenarios	References
Co-precipitation method	1. Simple and quick operation 2. Low cost, high yield, suitable for large-scale production	1. Poor shape control 2. Wide particle size distribution 3. Prone to oxidation	Fe_3_O_4_, γ-Fe_2_O_3_, MnFe_2_O_4_	Large-scale Engineering Applications for Sludge Leachate Treatment	[15,36]
Pyrolysis method	1. Excellent monodispersity 2. Precise control over dimensions and morphology 3. High crystallinity, superior magnetic properties	1. High temperatures, harsh reaction conditions 2. High cost, use of toxic reagents 3. The product is oil-soluble and requires functionalization to convert to an aqueous phase	Fe_3_O_4_, FePt	Small-batch laboratory production of precision magnetic materials	[37,38]
Hydrothermal/Solvent-thermal method	1. Good crystallinity, high purity 2. Capable of producing complex and specialized morphologies	1. Long reaction cycle 2. Unable to observe the reaction process in real time 3. High safety requirements for reactors	Various ferrites, particles with special morphologies	Laboratory Research on High-Performance Magnetic Cores	[28]
Microemulsion Method	1. Particle size is uniform and easy to control 2. Micro-reactors effectively prevent agglomeration	1. Extremely low yield 2. High surfactant consumption and cumbersome post-treatment processes 3. High cost	Fe_3_O_4_ CoFe_2_O_4_	Small-scale production of ultra-precise nanomaterials	[39,40]
Mechanical Ball Milling Method	1.Simple process 2.Large output	1. Wide particle size distribution, irregular morphology 2. Prone to introducing impurities and defects	Metal Alloy powder	Low-performance, resource-light applications	[41]

### 3.2. Surface Functionalization Modification Strategy

Magnetic nanoparticles (such as Fe_3_O_4_ and MnFe_2_O_4_) exhibit outstanding physicochemical properties, including biocompatibility, low toxicity, good electrical conductivity, chemical stability, and strong adsorption and catalytic capabilities toward heavy metal ions (HMIs). Characterized by high specific surface area, they are highly responsive to redox processes, pH changes, or the effects of corrosive reagents [42]. However, pure Fe_3_O_4_ is unstable in acidic environments and readily oxidizes into maghemite nanoparticles, resulting in a decline in performance. Therefore, surface functionalization strategies such as inorganic coating, carbon composite materials, organic polymer grafting, functional molecule impregnation, and metal–organic framework (MOF) composites are required to enhance its stability, dispersibility, and functionality [18,43]. Table 2 summarizes the comparative analysis of different functionalized magnetic materials.

Among various surface modification techniques, inorganic coating serves as a widely adopted and effective method for surface functionalization. It offers advantages such as low raw material costs, a simple process, minimal equipment requirements, and ease of scaling up. By encapsulating magnetic materials with an inorganic protective shell composed of substances such as SiO_2_ or MnO_2_, this approach not only significantly enhances material stability but also introduces additional functional groups [44]. This endows the coated materials with superior application prospects in the treatment of sludge leachate. Chitosan exhibits poor mechanical strength and solubility under acidic conditions, limiting its reusability. Conversely, silica’s high mechanical and thermal stability significantly improves the composite material’s resistance to swelling and mechanical properties [45]. Alves et al. demonstrated that silica-coated chitosan maintains greater structural stability in acidic and alkaline environments. Furthermore, crosslinking can further enhance bonding strength, ensuring material integrity during adsorption and improving reuse efficiency [46]. In applications demanding the lowest possible cost and high physical stability, inorganic coating represents a highly desirable solution. However, this method also has notable limitations: the types of functional groups in inorganic coatings are limited, their selectivity for adsorbing specific heavy metals is poor, and if the coating thickness is not properly controlled, it may reduce the material’s saturation magnetization, thereby affecting the efficiency of magnetic separation.

In contrast to the low-cost nature of inorganic coating, carbon-based composite modification focuses more on enhancing the material’s adsorption capacity and electrical conductivity. This is achieved by physically or chemically bonding the target material with carbon-based materials (such as graphene and CNTs) to form a synergistic system, thereby improving the adsorption of heavy metals from sludge leachate. However, its raw material costs are high (especially for high-quality graphene and CNTs), the manufacturing process is relatively complex, and energy consumption is high. Guo et al. achieved composite modification of magnetic components (NiFe_2_O_4_) with carbon-based materials (chitosan CS, graphene oxide GO) via a simple hydrothermal method. It can function stably in aqueous solutions and maintains structural and performance stability even after composite formation with chitosan and graphene oxide, supporting multiple adsorption cycles for reuse. Through component dispersion control, chemical interactions, and heat treatment, they formed NFCG composite materials exhibiting both high adsorption performance and magnetic recovery capability. Here, C=O, C-O, O-H, and aromatic groups contribute to Co (II) adsorption by forming Co-O bonds. At pH 7, the adsorption efficiency for Co(II) reached 96.87% [47]. In addition, the combination of chitosan and biochar can enhance adsorption performance; however, the pore structure and functional group content of the biochar directly affect the effectiveness of the composite. If the raw materials are not properly pretreated, it can easily lead to particle agglomeration, which in turn reduces adsorption and separation performance [48]. If the budget allows, carbon-based composites are an ideal choice for applications requiring high electrical conductivity, good adsorption properties, or catalytic support functions; however, their high cost and complex manufacturing processes remain major limiting factors.

Grafting of organic polymers is the preferred approach for balancing the introduction of specific functional groups with cost-effectiveness, and it is also one of the most environmentally friendly modification methods. By grafting organic polymers such as chitosan derivatives, polypyrrole (PPy), and polyaniline (PANI), or functional groups such as EDTA and quaternary ammonium ions onto the surface of magnetic materials, the adsorption selectivity and dispersibility of the materials can be precisely controlled [49]. Fe_3_O_4_/PPy/PA nanocomposites use PPy as a scaffold and are doped with phosphoric acid (PA), enabling synergistic adsorption of dyes and heavy metals through electrostatic attraction [50]. The Fe_3_O_4_/SiO_2_/PANI-SDBS composite adsorbs Pb (II) and Cd (II) through functional group interactions between PANI and SDBS. At pH = 7, the Pb(II) removal rate reaches 94.10%, while the Cd (II) removal rate achieves 77.47% [51]. The advantages of this method include high functional group tunability, moderate cost, and a relatively simple process; however, if the polymer grafting density is too high, it may cover the active sites on the surface of the magnetic material, potentially reducing the material’s saturation magnetization and thereby affecting its magnetic separation performance. Furthermore, some polymers are prone to degradation during long-term treatment of sludge leachate, leading to the loss of functional groups and reducing the material’s reusability.

The functional molecule impregnation method is the simplest modification technique to perform and requires the least capital investment. Its core advantages lie in its simplicity and short processing time, enabling rapid functional group modification of material surfaces; it is particularly suitable for the targeted treatment of specific heavy metals. The cost of this method depends primarily on the functional molecules themselves. By impregnating materials with small molecules such as APTES, EDTA, or specific acids and bases, targeted functional groups can be introduced onto the material surface: APTES-functionalized magnetic iron oxide nanoparticles (MIONPs) prepared via reflux carry amino groups (MIONPs-NH_2_) and exhibit high affinity for Pb (II) [52]. HNO_3_ modification introduced nitro and carboxyl groups, while KOH increased hydroxyl groups on the biochar. KOH-BC300 exhibited adsorption capacities of 72.14 mg/g for Cd (II) and 170.84 mg/g for Pb(II) [53]. Acidic chloraluminate ionic liquid (IL) impregnation of MIL-100(Fe) enhances Cr (VI) adsorption [54]. However, a critical drawback of this method is poor binding stability. The binding between small-molecule functional groups and the material surface is primarily physical adsorption, which is prone to detachment during the agitation and elution of sludge leachate, leading to a decline in adsorption performance. Furthermore, there has been limited research on the impact of this method on the magnetic properties of the material, making it difficult to guarantee the magnetic separation and recovery efficiency of the modified material. As such, this method is only suitable for rapid prototyping or the loading of expensive biomolecules; for practical large-scale applications, the issue of binding stability must first be resolved.

MOF composites are a specialized choice for high-end applications, with their core advantages lying in their ultra-high specific surface area, precise pore structure, and excellent adsorption selectivity. By integrating MOFs with magnetic materials, SiO_2_, and other substrates, it is possible to simultaneously improve the material’s dispersion, stability, and adsorption performance. However, this approach has significant limitations: raw materials such as MOF ligands are expensive, and the preparation process is complex and energy-intensive, making it difficult to scale up for widespread application. Fe_3_O_4_@SiO_2_-UiO-66-EDTA incorporates EDTA into UiO-66, combining the structural stability of UiO-66 itself under harsh acidic and alkaline conditions, the SiO_2_ shell prevents Fe_3_O_4_ agglomeration and framework collapse, and remains firmly attached after EDTA grafting. providing additional coordination sites and improving thermal stability [55]. NH_2_-MIL-101(Fe) was grown in situ on Fe_3_O_4_@mSiO_2_, forming a stable hybrid structure via electrostatic interactions between silicon dioxide chlorine groups and the MOF. Water-resistant, acid- and alkali-resistant with excellent thermal stability. The crystal structure remains largely intact after multiple adsorption–desorption cycles, exhibiting low Fe ion leaching and stable performance when treating organic pollutants in aqueous solutions [56]. Simonescu et al. employed mesoporous silica coating in Fe_3_O_4_@mSiO_2_/NH_2_-MIL-101(Fe) composites, providing a high-surface-area scaffold for uniform MOF growth while protecting magnetite nanoparticles [57]. This method is suitable only for scenarios requiring ultra-high adsorption performance, smart response characteristics, or cutting-edge applications; however, its high cost means it cannot replace the first four methods for large-scale implementation.

**Table 2:** Comparative Analysis of Different Functionalized Magnetic Materials.

Adsorbent	Functionalization Strategy	Synthesis Method	Primary Functional Group	Target Heavy Metals	Optimal pH	Adsorption Capacity (mg/g)	Adsorption Efficiency	References
CS/BC	Inorganic coating	one-pot synthesis	-NH_2_, -COOH, -OH	Hg (II)	3.0	594	80%	[58]
NiFe_2_O_4_	Carbon-based composite materials	Hydrothermal method	C=O, C-O, O-H, Aromatic group	Co (II)	9.0	387	96%	[47]
Fe_3_O_4_/SiO_2_/PANI-SDBS	Organic polymer grafting	Sol–gel method	Fe-O, Si-O-Si, N-H, C=C, S=O	Pb (II) Cd (II)	7.0	72 67	94%	[51]
KOH-BC300	Functional Molecular Impregnation	Surface complexation	-OH	Pb (II) Cd (II)	5.0	170 72	--	[53]
NH_2_-MIL-101(Fe)	Metal–Organic Framework Composites	Solvent-thermal method	C=C, -COOH, -OH, Fe-O	Pb (II)	5.5	150	92%	[56]

In summary, each functionalization strategy has distinct advantages and limitations. A comparison of key performance metrics for different surface functionalization strategies is shown in Table 3. Future research must address two key challenges: first, optimizing the modification process to enhance adsorption performance while minimizing the impact on the material’s saturation magnetization, thereby ensuring efficient recovery of the material from viscous sludge media; second, strengthening the synergistic application of different methods to reduce the cost of high-end approaches and improve the selectivity and stability of low-cost methods, thereby expanding the practical application potential of magnetic materials in sludge leachate treatment.

## 4. Characterization and Adsorption Mechanism of Heavy Metals in Sludge Leachate Using Magnetic Adsorbents

### 4.1. Advanced Characterization Techniques for Adsorption Mechanisms

Advanced characterization techniques are crucial for elucidating the adsorption mechanism of magnetic adsorbents toward heavy metals in sludge leachate. X-ray photoelectron spectroscopy (XPS) is widely employed to analyze surface chemical states and interactions: XPS spectra of chitosan-modified biochar after Cr (VI) adsorption revealed peaks at C–Cr (287.1 eV) and [Cr(CO)_6_(s)] (287.9 eV), directly demonstrating the involvement of surface functional groups. Similarly, Wang et al. and Liu et al. combined XPS and Fourier Transform Infrared Spectroscopy (FTIR) to confirm key mechanisms: -C=O and C-O groups were identified as the primary functional groups in adsorption, while XPS and FTIR results revealed electrostatic adsorption and π-π stacking as the main mechanisms [49,61].

FTIR is essential for identifying functional groups. Youssif et al. used FTIR characterization of the Fe_3_O_4_/SiO_2_/PANI-SDBS nanocomposite, which confirmed specific chemical bonds at peaks at 575 cm^−1^ (Fe-O stretching), 1088 cm^−1^ (Si-O), and 3222 cm^−1^ (N-H vibration in PANI. Scanning electron microscopy (SEM) provided insights into surface morphology [51]. Lohan et al. observed that pristine biochar (BC) exhibited a smooth, layered porous structure, while magnetic biochar (MBC) displayed smaller pore channels and metallic clusters. Chitosan coating thickened the surface, and EDTA modification increased surface roughness with more agglomerates—all factors relevant to the availability of adsorption sites [43]. Youssif & Wojnicki further employed SEM, transmission electron microscopy (TEM), BET specific surface area analysis, and X-ray diffraction (XRD). XRD confirmed the cubic spinel crystal structure of Fe_3_O_4_, exhibiting characteristic peaks at 2θ = 30.04°, 35.32°, and others, verifying crystallinity [51].

**Figure 2:**
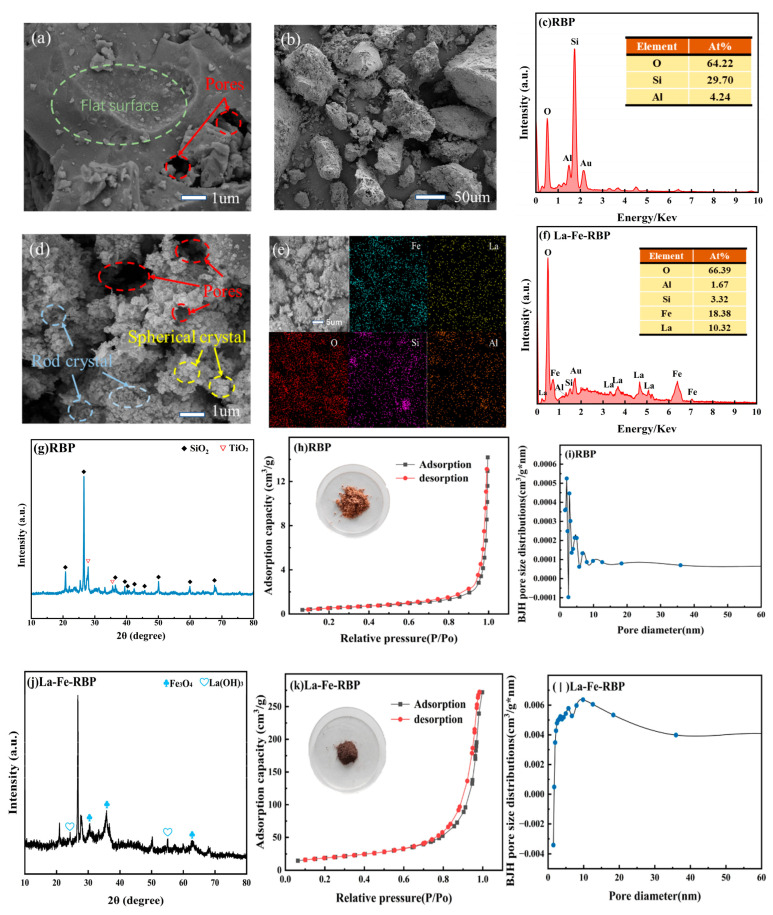
SEM images, EDS data, XRD patterns, N2 adsorption–desorption isotherms, and BJH PSDs of RBP (**a**–**c**,**g**–**i**) and La-Fe-RBP (**d**–**f**,**j**–**l**). Image courtesy of [62].

Zhao et al. further confirmed through XRD and BET characterization (Figure 2) that the modified La-Fe-RBP retained a mesoporous structure conducive to mass transfer, while also exhibiting a rough, porous surface rich in uniformly distributed Fe and La active sites. This structural characteristic not only provides ample contact interfaces and pathways for the efficient adsorption of pollutants but also endows the material with excellent magnetic separation performance, thereby validating the feasibility and superiority of using inexpensive waste red brick powder to construct high-efficiency magnetic adsorbents [62].

XAFS + XPS can precisely identify key active sites, providing clear targets for the functionalization of magnetic adsorbents and addressing the issue of poor adsorption selectivity in complex matrices. Jin et al. employed a combination of XAFS and XPS to elucidate the microscopic mechanism of Ni (II) adsorption on magnetic graphene oxide (MGO). XAFS revealed that Ni (II) forms a stable bidentate coordination bond with -COOH and -OH groups on the MGO surface, which serves as the core active unit of the adsorption process; XPS confirmed significant shifts in the C=O and O-H peak positions following adsorption, further validating the involvement of these functional groups in the coordination. Based on the site-specific identification results, MGO was subjected to carboxylation modification to increase the surface -COOH density, resulting in a 42% increase in the material’s adsorption capacity for Ni (II) and a 35% improvement in selectivity even under the interference of co-existing ions in sludge leachate [63].

The in situ XRD-XAS coupling technique can address the issue of structural instability in magnetic adsorbents during long-term use in complex wastewater, providing a basis for the development of industrial-grade stable materials. Lee et al. used in situ XRD-XAS coupled analysis to investigate the structural stability of MnFe_2_O_4_/magnetic biochar (MBC) in sludge leachate. Real-time in situ XRD monitoring revealed that pure MnFe_2_O_4_ is prone to lattice distortion in acidic leachate; XAS analysis confirmed that Fe exists as Fe (III) and Mn as Mn (II), with partial ion leaching identified as the primary cause of performance degradation. To address structural defects, a mesoporous SiO_2_ coating strategy was employed to restrict the leaching of the magnetic core. As a result, after 10 cycles in sludge leachate at pH 3–8, the material retained 92% of its crystal structure integrity, with no significant decline in magnetic separation efficiency [64].

X-ray Absorption Fine Structure (XAFS) spectroscopy is an advanced technique for analyzing the local atomic and electronic structures of materials. By analyzing XAFS spectral features and structural parameters, Jin et al. revealed the adsorption mechanism of Ni (II) on MGO at the microscopic level, elucidating its binding mode and thereby providing crucial structural evidence for MGO’s high-efficiency adsorption performance [63]. These advanced characterization techniques, when combined, enable a comprehensive and in-depth understanding of the mechanisms by which magnetic materials adsorb heavy metal ions, providing a solid theoretical foundation for the design and application of magnetic materials.

### 4.2. Analysis of Primary Adsorption Mechanisms

**Figure 3:**
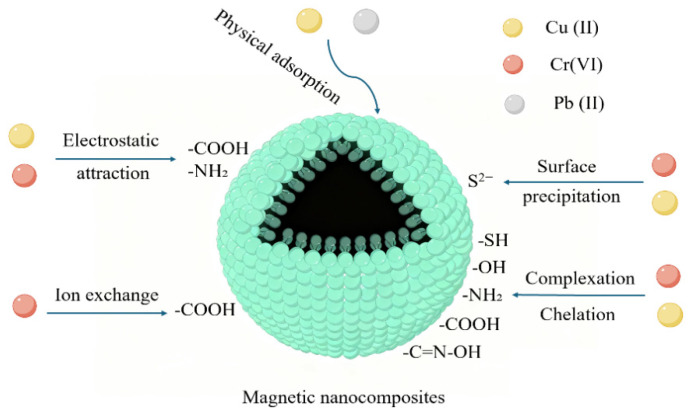
Adsorption mechanism diagram of magnetic nanocomposites.

Magnetic adsorbents used for removing heavy metals from sludge leachate exhibit multiple adsorption mechanisms, including electrostatic attraction, complexation and chelation, ion exchange, physical adsorption, and surface precipitation [43]. The contribution of these mechanisms is highly dependent on the physicochemical properties of the adsorbent (Figure 3). Electrostatic attraction plays a crucial role in heavy metal removal within weakly alkaline adsorbents. At pH = 2.0, the maximum adsorption capacity reached 492.61 mg/g, whereas at pH = 7.0, the adsorption capacity was only 90.5 mg/g—a decrease exceeding 80% from its peak value. This directly demonstrates the strong pH dependence of electrostatic attraction. In contrast, within strongly alkaline adsorbents, ion exchange becomes the dominant mechanism and exhibits minimal pH sensitivity [65]. Zhao et al.’s research provides corroborating evidence: the adsorption of Cu (II) by Fe_3_O_4_@SiO_2_-UiO-66-EDTA occurs primarily through electrostatic attraction. When the solution pH < 5.45, the Fe_3_O_4_@SiO_2_-UiO-66-EDTA surface carries a positive charge, leading to electrostatic repulsion with Cu (II) (cations). As pH decreases from 5 to 2, the removal efficiency of Cu (II) significantly declines; At pH > 5.45, the material surface becomes negatively charged, binding Cu (II) through electrostatic attraction. At pH = 5, Cu (II) adsorption approaches saturation, while at pH = 7, the Cu (II) removal rate increases to 95%, directly demonstrating that electrostatic attraction is the dominant mechanism for Cu (II) adsorption. Similarly, Cr (VI) undergoes the same electrostatic attraction process after being reduced to Cr (III) [55].

Beyond electrostatic interactions, complexation and chelation represent another significant category of chemical adsorption pathways. Their fundamental mechanism involves the formation of stable coordination bonds between functional groups on the adsorbent surface and heavy metal ions. The Fe_3_O_4_@SiO_2_-UiO-66-EDTA composite achieved effective removal rates of 95.52% for Cu (II) and 88.24% for Cr (VI) through chelation [55]. Regarding the adsorption of Co (II) by NFCG, spectral analysis confirms that the C=O, C-O, -C(=O)NH-, O-H, and aromatic groups on its surface provide lone pair electrons to form stable Co-O coordination bonds with Co (II). The removal rate for Co(II) reached 96.87%, with a corresponding adsorption capacity of 387.48 mg/g [47].

Meanwhile, physical adsorption serves as a fundamental mechanism that is widely prevalent, driven by factors such as van der Waals forces and pore trapping. Chen et al. observed that physical bonding is the primary force during the initial stage of pollutant adsorption on ceramic aggregates. Pollutant molecules first enter the material’s pores and are subsequently attracted and deposited by surface anionic groups [66]. Moreover, the adsorption of Cu (II) and Pb (II) by chitosan is also primarily governed by physical forces [67]. Under suitable conditions, certain heavy metal ions can also be immobilized through surface precipitation. Yang et al. studied GC_10_ and found that Cd (II) and Pb (II) preferentially react with CaS to form PbS and CdS precipitates. Excess Cd (II) can further form Cd-iron oxide complexes, while excess Pb (II) can form PbSO_4_. GC_10_ achieves removal rates exceeding 99% for Cd (II) and Pb (II), approaching near-complete removal. The quasi-second-order kinetic characteristics of this process indicate that chemical adsorption is the rate-limiting step throughout the adsorption process [68].

## 5. Performance Evaluation of Magnetic Adsorbents

### 5.1. Core Performance Metrics

Magnetic adsorbents for removing heavy metals from sludge leachate can be systematically evaluated based on four core indicators: adsorption capacity, adsorption kinetics, selectivity, and magnetic separation efficiency. Adsorption capacity is the fundamental parameter measuring the maximum adsorption capacity per unit mass of adsorbent for heavy metals, varying depending on the adsorbent and target metal. Guo et al. demonstrated that NFCG exhibits an adsorption capacity of 387.48 mg/g for Co(II) at an initial concentration of 200 mg/L and a dosage of 500 mg/L [47]. MnFe_2_O_4_/D201 exhibits a maximum adsorption capacity of 35.8 mg/g for As(V) at pH = 3 [31]. Fe_3_O_4_@SiO_2_-UiO-66-EDTA exhibits maximum adsorption capacities of 212.10 mg/g for Cu (II) and 118.10 mg/g for Cr (VI) [55]. The Fe_3_O_4_/SiO_2_/PANI-SDBS nanocomposite exhibits adsorption capacities of 72.20 mg/g for Pb(II) and 67.84 mg/g for Cd(II) at pH = 7 [51]. In addition, the adsorption capacities of Pst-MIMCl and CTS-GTMAC are 104.0 mg/g and 233.1 mg/g, respectively [65]. Even when treating organic dyes, PFSC exhibits maximum adsorption capacities of 955.0 mg/g for Orange Yellow II, 1075.8 mg/g for Acid Red 88, and 567.5 mg/g for Red Amaranth at pH = 3.0 [49].

Adsorption kinetics describes the rate and mechanism of adsorption, typically evaluated using kinetic models. It is important to clarify that the fitting of kinetic models merely provides an analytical tool for hypothesizing adsorption mechanisms. The quality of the fit does not directly prove the validity of the adsorption mechanism; it only offers a reference for the rate characteristics of the adsorption process. Research by Wang et al. indicates that both the adsorption of phosphates by Mg-Al-La LDH/Fe_3_O_4_/C and the adsorption of Hg by SP better conform to the pseudo-second-order kinetic model (R^2^ = 0.9996 for Hg adsorption, significantly higher than the R^2^ = 0.9262 for the pseudo-first-order model) [69,70]. Similarly, Zhao et al. found that the adsorption of Cu (II) and Cr (VI) on Fe_3_O_4_@SiO_2_-UiO-66-EDTA follows a pseudo-second-order model [55]. The adsorption of Pst-MIMCl and CTS-GTMAC also follows this model, and the adsorption of Pb (II) and Cd (II) on the Fe_3_O_4_@SiO_2_-UiO-66-EDTA/SiO_2_/PANI-SDBS nanocomposite similarly adheres to this model [51]. These cases merely reflect the fitting results under specific systems and should not be used to infer that pseudo-second-order kinetic models are more applicable across all adsorption systems.

Selective adsorption reflects an adsorbent’s preferential adsorption tendency toward different metals, typically under conditions where the target metal ion coexists with other competing ions. Zhao et al.’s research indicates that Fe_3_O_4_@SiO_2_-UiO-66-EDTA exhibits a higher adsorption capacity for Cu (II) (212.10 mg/g) than for Cr (VI) (118.10 mg/g), while the Fe_3_O_4_/SiO_2_/PANI-SDBS nanocomposite exhibited a higher adsorption capacity for Pb(II) (72.20 mg/g) than for Cd(II) (67.84 mg/g) [51,55]. Magnetic separation efficiency is a key performance metric for evaluating the recovery and reuse potential of adsorbents, closely related to their superparamagnetic properties and saturation magnetization. Youssif et al. reported saturation magnetization values of 12.5 and 10.5 emu/g for Pst-MIMCl and CTS-GTMAC, respectively. Due to their superparamagnetic characteristics, these materials exhibit effective magnetic separation efficiency [51]. Guo et al. Through experiments, it is found that the mass ratio of NiFe_2_O_4_ in NFCG is 61.44%, and the high content of magnetic components ensures sufficient saturated magnetic moment, which provides a material basis for magnetic separation. NFCG is also recovered efficiently by paramagnetism [47].

### 5.2. Cyclic Stability of Magnetic Adsorbents

The cycling stability of magnetic adsorbents is a key indicator for evaluating their reuse potential, with significant variations observed among different materials. Zhao et al. experimentally demonstrated that Fe_3_O_4_@SiO_2_-UiO-66-EDTA maintained excellent adsorption performance after 10 cycles, achieving removal efficiencies of 80.89% for Cu (II) and 81.86% for Cr (VI) [55]. Similarly, Benjedim et al. found that the magnetic adsorbents Fe_3_O_4_ + C and CoFe_2_O_4_ + C exhibited no significant decrease in adsorption capacity for Pb (II) and Cd (II) after the first cycle (*p* < 0.01). The adsorption capacities of Fe_3_O_4_ + C for Pb (II) and Cd (II) were 389 mg/g and 269 mg/g, respectively, while CoFe_2_O_4_ + C exhibited adsorption capacities of 249 mg/g and 264 mg/g for Pb (II) and Cd (II), respectively, outperforming many previously reported materials [71].

It is important to critically note that current discussions on the cycling stability of magnetic adsorbents often focus on the decay patterns of adsorption performance while overlooking the practical feasibility of the regeneration process. Magnetic adsorbents are typically categorized as low-cost materials, with one core application being the reduction in treatment expenses. However, the regeneration process often consumes chemical reagents and energy while potentially generating secondary pollution. From both economic cost and environmental impact perspectives, its feasibility can sometimes fall below that of direct disposal. This research orientation of “regeneration for regeneration’s sake” fails to adequately integrate practical engineering scenarios, potentially hindering the translation of laboratory-level recycling performance advantages into tangible application value.

However, some adsorbents exhibit performance degradation during cycling, primarily attributed to strong interactions between heavy metal ions and surface functional groups. This prevents complete elution during regeneration, causing partial occupation of active sites. To mitigate this issue, researchers enhanced material stability through structural modification. Yang et al. developed the composite material GC10 by co-pyrolyzing TiG with corn cobs. Its structure incorporates gypsum, calcium sulfide, and magnetic iron oxide, achieving a saturation magnetization of 6.3 emu/g, facilitating magnetic separation. demonstrated over 99% removal efficiency for Cd (II) and Pb (II). Compared to pristine TiG, the adsorption kinetic constant (k1) significantly increased (218-fold for Cd (II) and 9-fold for Pb (II)), indicating substantial enhancement in both cycling stability and reuse potential [68]. However, such modification methods must still balance the feasibility of the regeneration process to avoid losing the cost advantage of inexpensive adsorbents due to increased modification costs.

### 5.3. Desorption of Magnetic Adsorbent

Desorption of magnetic adsorbents depends on selecting suitable desorbents, understanding its mechanism and optimizing process parameters in order to realize effective heavy metal release and adsorbent reuse. Common desorbents include NaOH solution, and its concentration is adjusted according to the properties of the adsorbent. Zhang et al. Desorption of As(V) from MnFe_2_O_4_/D201 composite adsorbent with 0.1 mol/L NaOH [31]. Strong alkaline magnetic adsorbents such as Pst-MIMCl need more stringent conditions. Sun et al. use 0.3 mol/L NaOH + 0.3 mol/L NaCl to desorb Cr (VI) from composite adsorbents, while weak alkaline adsorbents can only desorb Cr (VI) with 0.1 mol/L NaOH [65]. The desorption mechanism corresponds to the adsorption mechanism: weakly basic adsorbents are desorbed by NaOH, which involves deprotonation of amino groups and transformation of chromium species, while strongly basic adsorbents relying on ion exchange need a NaOH + NaCl mixture [65].

Optimizing the type and concentration of desorption agent can improve efficiency and reusability. The desorption efficiency of MnFe_2_O_4_/D201 reached 81.3% after ten cycles using 0.1 mol/L NaOH [31]. However, the desorption efficiency of Pst-MIMCl using mixed desorbents reaches 99.2%, and it can be reused in ten cycles [65]. In addition, the step-by-step desorption process can effectively separate and recover the adsorbed heavy metals, and the desorption residue can be recovered by pressurized alkali leaching for P-CSH synthesis [72]. These key performance indicators comprehensively evaluate the performance of magnetic materials in adsorbing and removing heavy metals from sludge leachate from various perspectives, providing important references for adsorbent selection and optimization.

## 6. Summary and Future Outlook

### 6.1. Summary

This review systematically summarizes recent research progress on the synthesis, functionalization, mechanisms, and performance of magnetic adsorbents for the removal of heavy metals from sludge leachate. Sludge leachate is characterized by a complex matrix featuring wide pH fluctuations, the coexistence of multiple metals, and interference from high organic matter and high salt content, which significantly influence adsorption behavior and material suitability. Based on the characteristics of the leachate, the following material selection recommendations are provided: In acidic systems, MnFe_2_O_4_, SiO_2_-coated, and MOF composite magnetic materials are preferred to enhance acid resistance and structural stability; In neutral and weakly alkaline systems, carbon-based composites and grafted organic polymer materials should be prioritized to strengthen electrostatic and complexation adsorption; cation-specific chelating functionalized adsorbents should be used for Pb (II), Cd (II), and Cu (II), while anion-specific alkaline and ion-exchange materials should be used for Cr (VI) and As (V); In high-organic environments, prioritize SiO_2_-coated and sieve-like porous materials to reduce competitive interference. Current research has achieved multidimensional analysis of adsorption mechanisms and improvements in material performance; however, bottlenecks remain, including insufficient adaptability to complex matrices, difficulties in large-scale synthesis, poor economic viability of regeneration, and a lack of long-term stability and safe disposal methods.

### 6.2. Future Outlook

Future research should focus on three urgent scientific and engineering challenges: First, developing highly selective adsorbents capable of functioning in the presence of high organic matter content to mitigate competitive adsorption by humic acids and ammonia nitrogen; second, establishing low-cost, environmentally friendly, and scalable large-scale production processes to facilitate the transition of laboratory findings into engineering applications; and third, enhancing long-term stability under actual operating conditions and conducting life-cycle assessments to ensure safe reuse and harmless disposal. By integrating computational modeling and machine learning-driven design to construct an integrated “materials–process–regeneration–disposal” solution, we can provide critical support for the sustainable management of heavy metal pollution in sludge leachate.

## Figures and Tables

**Table 3:** Comparison of Key Performance Characteristics of Different Surface Functionalization Strategies.

Functionalization Strategy	Effects on Magnetic Stability	Adsorption Capacity Contribution	Heavy Metal Selectivity	Cost of Raw Materials	Scalability	Stability of Real Sludge Media	Factors Affecting Magnetic Separation Efficiency	References
Inorganic coating	Significantly improved, acid and alkali-resistant	Low–Medium	Low	Low	Good	Good	Slight decrease	[59]
Carbon-based composite materials	Medium, The carbon layer provides protection	High	Medium	Medium (Graphene)	Medium (Biochar)	Medium	Remains largely unchanged	[60]
Organic polymer grafting	Medium, Depending on the degree of cross-linking	Medium–High	High	Medium	Medium	Medium–Bed (Acidic and prone to swelling)	Remains largely unchanged	[51]
Functional Molecular Impregnation	Low, May etch the magnetic core	Medium	Medium	Low	Good	Bed (Easy to wash out)	Remains largely unchanged	[53]
MOF composite	High, Scaffold protection	Polar altitude	Polar altitude	High	Bad	Medium (The channels are prone to clogging)	Slight decrease	[56]

## Data Availability

No new data were created or analyzed in this study. Data sharing is not applicable to this article.
